# Functional and pharmacological characterization of the *SCN2A* variant p.C258R with mixed gain and loss of function causing developmental and epileptic encephalopathy type 11

**DOI:** 10.1016/j.ebr.2026.100897

**Published:** 2026-09-11

**Authors:** Alina Köppel, Heike de Vries, Mahdi Jamili, Christina Stanke, Ulrich Brandl, Enrico Leipold, Stefan H. Heinemann

**Affiliations:** a Center for Molecular Biomedicine, Department of Biophysics, Friedrich Schiller University Jena and Jena University Hospital, Jena, Germany; b Department of Neuropediatrics, Jena University Hospital, Jena, Germany; c Department of Anesthesiology and Intensive Care, University Hospital Schleswig-Holstein, Center for Brain, Behavior and Metabolism (CBBM), University of Luebeck, Germany

**Keywords:** Sodium channel, Na_V_1.2, Developmental epileptic encephalopathy, Antiseizure medication, Sodium channel block, Patch clamp

## Abstract

Pathogenic variants in *SCN2A* cause dysfunction of the Na_V_1.2 voltage-gated sodium channel and are associated with neurodevelopmental disorders with or without epilepsy. Treatment of epilepsy in these patients is challenging and depends on whether the variant results in a gain (GoF) or loss of function (LoF). We describe a patient with the *de novo SCN2A* variant p.C258R presenting with therapy-resistant developmental and epileptic encephalopathy type 11 (DEE type 11). We aimed to electrophysiologically analyze the functional consequences of this mutation and to examine the *in vitro* properties of phenytoin, lamotrigine, and topiramate, which resulted in clinical improvement. Depending on the specific functional readout after transient expression in HEK293T cells, mutation C258R of Na_V_1.2 channels resulted in a mixed LoF and GoF phenotype. Na_V_1.2-C258R channels exhibited slower inactivation kinetics and a left-shifted voltage dependence of steady-state inactivation. Current density was reduced when expressed in HEK293T cells although single-channel conductance and maximal channel open probability were only marginally affected. During repetitive stimulation, mutated channels showed stronger cumulative inactivation, consistent with LoF. Phenytoin and lamotrigine caused use-dependent block, but lamotrigine was less effective for C258R compared with the wild type. Topiramate diminished the current density in both variants with less use-dependence. The C258R mutation impairs Na_V_1.2 function through both LoF and GoF mechanisms. Sodium channel blockers with a strong use-dependence, such as phenytoin, may be preferential for seizure control in such cases.

## Highlights

• Mutation p.C258R in *SCN2A* causes DEE type 11.
• The mutation, located in DI S5 of sodium channel Na_V_1.2, results in slower inactivation but overall lower channel activity.
• Sodium-channel blocker show variable efficacy, depending on their mechanism of action.

## 1 Introduction

Epileptic disorders are among the most common chronic diseases in childhood, with up to 1% of children being affected [1]. In epilepsy, abnormal and excessive neuronal activity in the brain's cortex provokes seizures. The imbalance between the excitatory and inhibitory signals of neurons can have both genetic and acquired causes. Most of the genes involved code for ion channel proteins and transporters. Commonly recognized origins of congenital epilepsy syndromes are variants of voltage-gated sodium (Na_V_) channels, predominantly missense mutations [2].

Na_V_ channels are essential for the initiation and propagation of action potentials, as they are responsible for the rapid voltage-gated influx of Na^+^ in excitable cells. Na_V_1.2, encoded by *SCN2A*, is highly expressed in the central nervous system, located mainly at the axon initial segment of myelinated (early development) or unmyelinated axons (adult brain). Other Na_V_ channel subtypes localized in central neurons are Na_V_1.1, Na_V_1.3 and Na_V_1.6, encoded by *SCN1A*, *SCN3A* and *SCN8A*, respectively [3]. Since even minor alterations in these genes cause remarkable effects in Na_V_ channel expression and molecular function, they lead to a plethora of neurological and psychiatric diseases [4], [5], [6], [7], [8], [9].

Although mutations in *SCN2A* are less frequent than *SCN1A* defects [2], [8], patients with inherited or *de novo* gene alterations in *SCN2A* are not uncommon [2], [10], [11]. The phenotypic spectrum ranges from mild cases, including self-limited familial neonatal-infantile seizures (the first entity in which *SCN2A* mutations were detected) and generalized epilepsy with febrile seizures [12], [13], [14], [15], [16], to severe epileptic disorders, including early-onset epileptic encephalopathies, severe myoclonic epilepsy of infancy (Dravet syndrome), early infantile epileptic encephalopathy with burst suppression (Ohtahara syndrome), infantile spasms (West syndrome), epilepsy of infancy with migrating focal seizures and further unclassified intractable epilepsies [11], [15], [17], [18], [19], [20], [21], [22], [23], [24], [25]. Inherited *SCN2A* mutations mainly lead to benign diseases such as self-limited familial neonatal infantile seizures, whereas severe phenotypes are predominantly caused by *de novo* missense or truncating mutations [13], [15], [21].

Defects in *SCN2A* were also associated with further clinical symptoms, suggesting the involvement of Na_V_1.2 channels in the etiology of autism spectrum disorder (ASD), intellectual disability, schizophrenia, and movement disorders [6], [8], [9], [17], [26], [27], [28], [29], [30]. Whereas variants related to ASD basically result in reduced or complete loss of Na_V_1.2-mediated Na^+^ current, the functional impact of infantile seizures-associated variants on neurons often remains unclear and may result in *gain-of-function* (GoF) (predicting an increase in neuronal excitability)*, loss of function* (LoF), or both [10], [11], [22], [26], [31]. Therefore, optimizing an effective treatment is not straightforward and often requires several medication attempts. Furthermore, it is essential to understand whether a given mutation might influence the efficacy of antiseizure medication (ASM, or anticonvulsants).

There is still no clear genotype-phenotype correlation among the disorders associated with *SCN2A* variants [10], [26]. Especially in cases with mixed GoF and LoF gating properties, functional characterization of *SCN2A* mutations is important for treatment optimization [30], [32]. While Na_V_ channel LoF is not readily accessible to pharmacological intervention, the treatment of GoF variants with Na_V_ channel blockers appears obvious. However, depending on the type of Na_V_ channel dysfunction and age of disease onset, Na_V_ channel blockers can even worsen seizure activity [11].

In this study, we examine a new early-onset epileptic encephalopathy caused by a *de novo* missense *SCN2A* mutation. We functionally analyze the electrophysiological properties by performing whole-cell patch-clamp recordings in human embryonic kidney 293 T cells and compare the results to cells expressing wild-type Na_V_1.2. In addition, we analyze the response of wild-type and mutant Na_V_1.2 channels to ASMs administered to the patient to clarify the correlation between the patient's treatment response and the efficacy of the prescribed drugs in blocking the channels.

## 2 Materials and methods

### 2.1 Anamnesis and diagnostics

The patient was examined in clinics in Germany specialized in epileptic and neurological disorders to determine the cause of his disease. The Center for Genomics and Transcriptomics in Tübingen analyzed samples of his genomic DNA. Mutation analysis of *SCN2A* (and further genes known to cause epileptic encephalopathies *inter alia SCN1A* and *KCNQ2*) was performed on all exons and exon-intron boundary regions by PCR and compared to a non-affected human reference DNA. Two unclassified gene modifications were identified: in *SCN2A* (c.772 T > C; p.C258R; p.Cys258Arg) and in *PLCB1* (1-phosphatidylinositol-4,5-bisphosphate phospholipase beta-1, c.1469 A > G; p.Y490C; p.Tyr490Cys). Both mutations have not been reported so far. According to prediction programs of the pathogenicity (Mutation Taster, PolyPhen-2, and Sorting Intolerant from Tolerant), the variation in *PLCB1* was categorized as benign, while the *SCN2A* defect was classified as pathogenic. The asymptomatic parents of the index patient did not harbor the respective mutations and, hence, *SCN2A* p.C258R is a *de novo* mutation.

The study was approved by the Jena University Hospital Institutional Review Board (study number 2025–4055-BO-D).

### 2.2 Expression constructs and cell transfection

The sequence of the human neonatal splice form of *SCN2A* (acc. no. NM_001040143, encoding the nNa_V_1.2 channel α subunit) was cloned into a pCIneo vector for expression in mammalian cells. Mutation p.C258R was created using the QuikChange Site-Directed Mutagenesis Kit (Stratagene) and verified by sequencing. The adult variants of Na_V_1.2 and Na_V_1.2-C258R were generated by introducing the mutation N209D. This site is located in the extracellularly accessible linker between transmembrane segments S3 and S4 in domain I, and it does not noticeably affect the overall gating parameters of the channels [33].

Human embryonic kidney 293 T cells (HEK293T, CAMR, Porton Down, Salisbury, UK) were cultured in 45% Dulbecco's Minimal Eagle's Medium (DMEM) and 45% Ham's F12 medium, supplemented with 10% fetal bovine serum in a humid 37 °C incubator with 5% CO_2_. Cells were transiently co-transfected with the respective channel-coding plasmids and a plasmid encoding CD8 using the Roti-Fect transfection kit (Carl Roth, Karlsruhe, Germany) according to the supplier instructions. Anti-CD8-coated Dynabeads (Deutsche Dynal GmbH, Hamburg, Germany) were used for visual identification of transfected cells. Electrophysiological recordings were performed 1–2 days after transfection.

### 2.3 Electrophysiology

Currents were recorded using the whole-cell patch-clamp configuration at 20 ± 1 °C with an EPC10 patch-clamp amplifier operated with PatchMaster software (both HEKA Elektronik, Lambrecht, Germany). Patch pipettes were fabricated from borosilicate glass (BioMedical Instruments, Zöllnitz, Germany) and were coated with silicone elastomer (RTV615 from KVD, Bad Wimpfen, Germany) or dental wax to reduce their capacitance. After fire-polishing the pipettes, resistances of 0.9–2.0 MΩ were obtained. Up to 85% of the series resistance was electronically compensated.

Data were sampled at a rate of 50 kHz after low-pass filtering with a cut-off frequency of 7 kHz. The holding voltage was −120 mV, and all current data shown were corrected for leak and capacitive currents using a p/6 protocol.

The pipette solution composed of (in mM): 35 NaCl, 105 CsF, 10 EGTA (ethylene glycol bis(2-amino-ethylether)tetraacetic acid), 10 HEPES (pH 7.4 with CsOH). The bath solution contained (in mM): 150 NaCl, 2 KCl, 1.5 CaCl_2_, 1 MgCl_2_, 10 HEPES (pH 7.4 with NaOH).

### 2.4 Chemicals and substance application

Lamotrigine, phenytoin, and topiramate were purchased from Sigma-Aldrich and stored at −20 °C as 100-mM stock solutions in DMSO. The ASMs were diluted in bath solution to their final working concentration immediately before experiments and were applied locally to cells with a glass pipette as described earlier [34].

### 2.5 Data analysis

Data were analyzed with FitMaster (HEKA Elektronik) and FITMASTER NEXT (Multi Channel Systems MCS GmbH, Reutlingen, Germany), and Igor Pro (WaveMetrics, Lake Oswego, OR, USA) using self-written code. If not stated otherwise, data are presented as means ± SEM (*n* = number of independent experiments). When appropriate, data groups were compared using unpaired Student's *t*-tests: *** *P* < 0.001; ** *P* < 0.01; * *P* < 0.05.

Channel activation. Voltage dependence and fast activation and inactivation kinetics were measured with step depolarizations between −80 and 60 mV in steps of 10 mV. The peak currents as a function of voltage were described according to a Hodgkin & Huxley formalism assuming three activation gates:

(1) $$I\left(V\right)=\Gamma \frac{1-e^{\left(-\left(V-E_{\mathrm{rev}}\right)/25\;\mathrm{mV}\right)}}{1-e^{\left(-V/25\;\mathrm{mV}\right)}}{\left(1+e^{-\left(V-V_{m}\right)/k_{m}}\right)}^{-3}$$

with the conductance Γ and the reversal potential *E*_rev_. *V*_m_ is the half-maximal activation voltage per gate and *k*_m_ the corresponding slope factor. To eliminate the impact of cell size, currents were normalized to the cell capacitance, *C*_m_.

Channel inactivation. Kinetics of channel inactivation between −50 and 0 mV were estimated by single-exponential fits. Closed-state inactivation between −90 and −60 mV was measured with a two-pulse protocol. Recovery from inactivation was also measured with a two-pulse protocol covering −120 to −80 mV. The entire voltage dependence of inactivation kinetics was described assuming a one-step reaction between non-inactivated and inactivated channels yielding the voltage-independent, limiting speed of inactivation *τ*_0_, as well as the maximal rate α_0_ at the half-maximal inactivation voltage *V*_h_:

(2) $$\tau \left(V\right)=\tau_{0}+{\left(\alpha_{0}e^{\left(V-V_{h}\right)\mathrm{q\delta}/\mathrm{kT}}+\alpha_{0}e^{-\left(V-V_{h}\right)q\left(1-\delta \right)/\mathrm{kT}}\right)}^{-1}$$

with the Boltzmann constant k, the absolute temperature *T*, the total gating charge transfer *q*, and the symmetry factor associated to fast channel inactivation δ.

Voltage dependence of steady-state inactivation was determined using prepulse protocols of either 500 ms (fast inactivation) or 10 s duration (slow inactivation) between −140 and −45 mV in steps of 5 mV or 10 mV, respectively. The resulting peak currents were plotted as a function of voltage and fit with a Boltzmann-type function:

(3) $$\frac{I\left(V\right)}{I_{-120\;\mathrm{mV}}}=h_{\max}-\left(h_{\max}-h_{\min}\right){\left(1+e^{-\left(V-V_{h}\right)/k_{h}}\right)}^{-1}$$

with the maximal and minimal channel availability *h*_max_ and *h*_min_, the half-maximal inactivation voltage *V*_h_, and the corresponding slope factor *k*_h_.

The response of the channels to repetitive depolarizations was measured with protocols in which depolarizations of 10 ms duration were elicited from a holding potential of −120 mV, varying the pulse frequency: 20, 33, 50, 66, 83 Hz.

Ramp protocols. From a holding potential of −120 mV, voltage ramps to 50 mV in 50, 100, and 200 ms were applied (*i.e.*, 3.4, 1.7, and 0.85 mV/ms). Ramp currents were corrected for linear leak and capacitive currents using a p/6 method with a leak holding of −130 mV and a leak size of 10%.

Non-stationary noise analysis. Single-channel current sizes were estimated with non-stationary noise analysis according to Steffan and Heinemann [35]. In brief, from a holding voltage of −120 mV, 20-ms pulses to −140, −100, −40, −30, −20, −10, 0, and 10 mV were applied. The first two pulses were used for linear leak and capacitive current correction. Pulses were separated by 100-ms episodes at −120 mV. 250 of such pulse trains were applied at 2 s intervals. To constrain the analysis, variance *versus* current at various voltages was fit simultaneously according to Heinemann and Leipold [36] to yield the single-channel current amplitude *i* and the maximal open probability *P*_open_ as a function of voltage.

## 3 Results

### 3.1 Clinical case report

The male index patient was born spontaneously after an uneventful pregnancy followed by an adequate development during the first months. He achieved unsupported sitting and crawling at the age of 1 year and walking at 1.5 years. However, his gait was impaired by marked ataxia; his fine motor skills were affected by tremor and dysmetria, and he exhibited muscular hypotonia. His cognitive development was severely delayed; no spoken language development was observed. He exhibited autistic features characterized by the absence of social laughter, avoidance of eye contact and physical proximity, minimal social interactions, and lack of interest in other children.

Apart from the mother's cousin, who had seizures in early childhood, the family history was negative for epilepsy or other neurological disorders. A comprehensive evaluation, including blood tests, metabolic screening, cerebrospinal fluid analysis, and MRI, were unremarkable; only a small parasagittal cyst was detected in cerebral CT. Audiological and ophthalmological examinations revealed normal function. The first bilateral tonic-clonic seizure occurred at the age of 23 months. From that time onward, he rapidly developed multiple focal and generalized seizure types such as tonic, myoclonic, bilateral tonic-clonic seizures, absences, nodding attacks, drop attacks, and apnea. Seizures occurred both during sleep and wakefulness. The duration of seizures was less than 1 min, with a frequency of 3 to 12 episodes per day. Seizures were also triggered by infections with fever. The EEG demonstrated moderate to severe generalized diffuse slowing of the background activity. Interictally, generalized and bilateral sharp waves and spike-waves were observed over the centro-parietal and frontal regions. In addition, multifocal slow sharp waves with a frequency of 1–2 Hz were present. The sleep EEG was abnormal, showing generalized polyspike-waves followed by amplitude depression. Ictally, during a generalized tonic-clonic seizure, the EEG demonstrated corresponding generalized low-voltage activity with fast rhythms, followed by rhythmic delta waves at a frequency of 2–3 Hz.

Rescue medications using diazepam, midazolam, or clonazepam were partially ineffective and seizures were even refractory to other GABAergic ASMs, such as valproate and phenobarbital. Levetiracetam, ethosuximide, and ketogenic diet were ineffective. The patient was treated three times with adrenocorticotropic hormone with only transient improvement in seizure frequency.

The previously administered ASM combined with topiramate had no substantial effect on seizure control. In contrast, the combination of topiramate (7 mg/kg/day) and lamotrigine (8 mg/kg/day) led to a significant reduction in seizure frequency, although lamotrigine required sequential dose escalation. Phenytoin was subsequently added as an adjunct to topiramate, effectively stabilizing seizure frequency and duration, reducing the need for rescue medication and allowing seizure-free intervals of several weeks. After a few years, an attempt to taper and discontinue topiramate resulted in seizure recurrence. Subsequent combinations of phenytoin with other GABAergic ASMs failed to restore prior seizure control. Only after reintroducing topiramate alongside phenytoin stabilized seizure frequency to the previously achieved controlled level.

Unfortunately, the medication only influenced the epileptic part of his syndrome. Developmental regression and motor retardation continued; speaking was still not possible, he needed help with all daily activities, including eating, drinking, personal care, and mobility. At the age of 3 years, he could no longer walk alone.

**Fig. 1:**
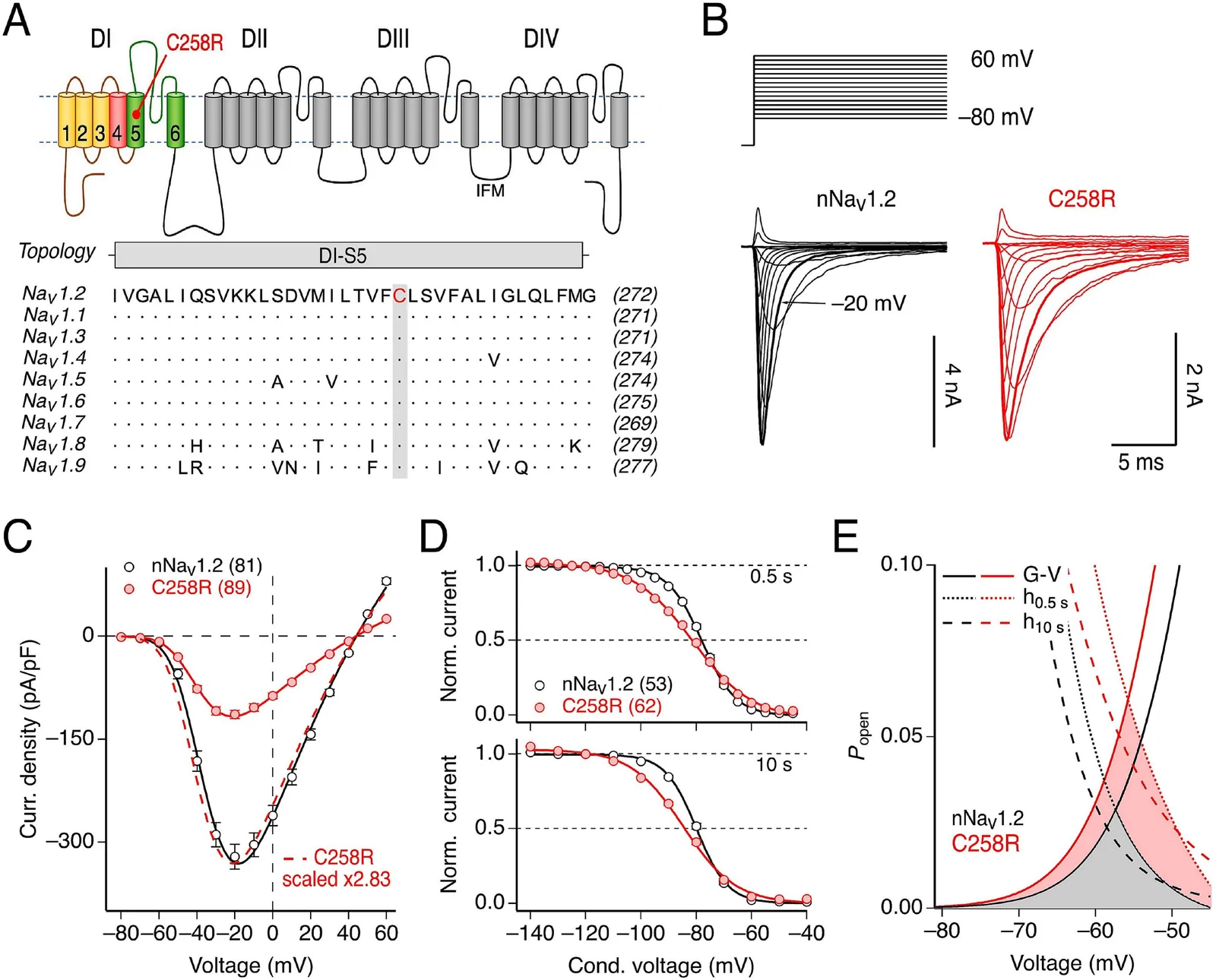
Voltage dependence of channel activation and inactivation. **A**) Membrane topology of a Na_V_1.2 α subunit with four homologous domains (DI-DIV) indicating the location of alteration p.C258R in the distal half of S5 in DI (*top*). The amino acid sequence alignment (*bottom*) indicates conservation of the cysteine residue among the nine human Na_V_ isoforms. Dots denote sequence conservation with respect to Na_V_1.2; numbers in parentheses refer to the last amino acid position shown. **B**) Superposition of whole-cell voltage-clamped currents from HEK293T cells expressing either nNa_V_1.2 wild type (black) or the mutant C258R (red), elicited with the indicated step depolarizations ranging from −80 to 60 mV. Thick traces correspond to pulses to −20 mV. **C**) Peak currents from recordings as in B), normalized to the cell capacitance as a function of voltage. The continuous curves are data fits according to eq. [1]. The red dashed curve is the result for mutant C258R, scaled by a factor of 2.83 to match the maximal current density obtained for the wild type. **D**) Mean maximal currents at −20 mV as a function of voltage of a 500-ms (*top*) or 10-s (*bottom*) prepulse, normalized to the values obtained at −120 mV. Curves are the results of Boltzmann-type fits (eq. [3]). Data in C) and D) are means ± SEM with *n* indicated in parentheses. **E**) Window current for nNa_V_1.2 and mutant C258R, estimated from the voltage-dependent activation (solid line) and steady-state inactivation with 0.5 s or 10 s conditioning (dotted and dashed lines, respectively). (For interpretation of the references to colour in this figure legend, the reader is referred to the web version of this article.)

At the age of 2.5 years, mutation analysis of his genomic DNA identified the heterozygous defect p.C258R in *SCN2A*, coding for the α subunit of voltage-gated sodium channel Na_V_1.2. The cysteine residue 258 is located in the transmembrane segment 5 of domain I (DI/S5) and is highly conserved among human Na_V_ channels (Fig. 1A).

### 3.2 Functional characterization of nNa_V_1.2-C258R

The functional impact of alteration p.C258R in neonate Na_V_1.2 (nNa_V_1.2) was analyzed by expressing wild-type and mutated sequences in HEK293T cells and performing whole-cell voltage-clamp experiments. Step depolarizations to various voltages from a holding voltage of −120 mV (to ensure almost complete recovery from inactivation) yielded typical voltage-gated Na^+^ currents (Fig. 1B). The averaged peak current densities were consistently smaller for mutant channels (factor of 2.83, Fig. 1C). The analysis of the voltage dependence of peak current densities with a Hodgkin & Huxley gating model (Fig. 1C, eq. [1]) yielded a half-maximal voltage for the activation of wild-type channel subunits of −46.6 ± 0.5 mV. The activation of C258R mutant channels was slightly but significantly shifted to more hyperpolarized potentials; they reached half-maximal activation at −50.2 ± 0.5 mV (*P* < 0.001). The associated slope factors characterizing the voltage dependence of activation were not different between wild-type and mutant channels (nNa_V_1.2: 11.4 ± 0.2 mV, C258R: 11.4 ± 0.1 mV).

The voltage dependence of steady-state fast inactivation was measured with 500-ms prepulses between −140 and −45 mV; the resulting test currents, normalized to the values at −120 mV, are shown in Fig. 1D (*left*). Fits according to a Boltzmann distribution (eq. [3]) yielded for wild-type channels a half-inactivation voltage (*V*_h_) of −77.8 ± 0.5 mV and an associated slope factor (*k*_h_) characterizing the voltage dependence of inactivation of 5.8 ± 0.1 mV. C258R mutant channels displayed a small left-shift in the half-inactivation voltage (−81.3 ± 0.6 mV, *P* < 0.001) as well as a significantly shallower voltage dependence (10.3 ± 0.2 mV, *P* < 0.001). Both aspects together have the consequence that at resting voltages (−100 to −80 mV) C258R channels tend to undergo more inactivation than the controls. Similar results were obtained for inactivation induced by long-lasting (10 s) prepulse depolarizations: *V*_h_ was −79.8 ± 0.4 mV and −84.7 ± 0.6 mV (*P* < 0.001), *k*_h_ was 5.3 ± 0.1 mV and 9.0 ± 0.1 mV (*P* < 0.001) for wild-type nNa_V_1.2 and C258R mutant channels, respectively (Fig. 1D, *right*).

**Fig. 2:**
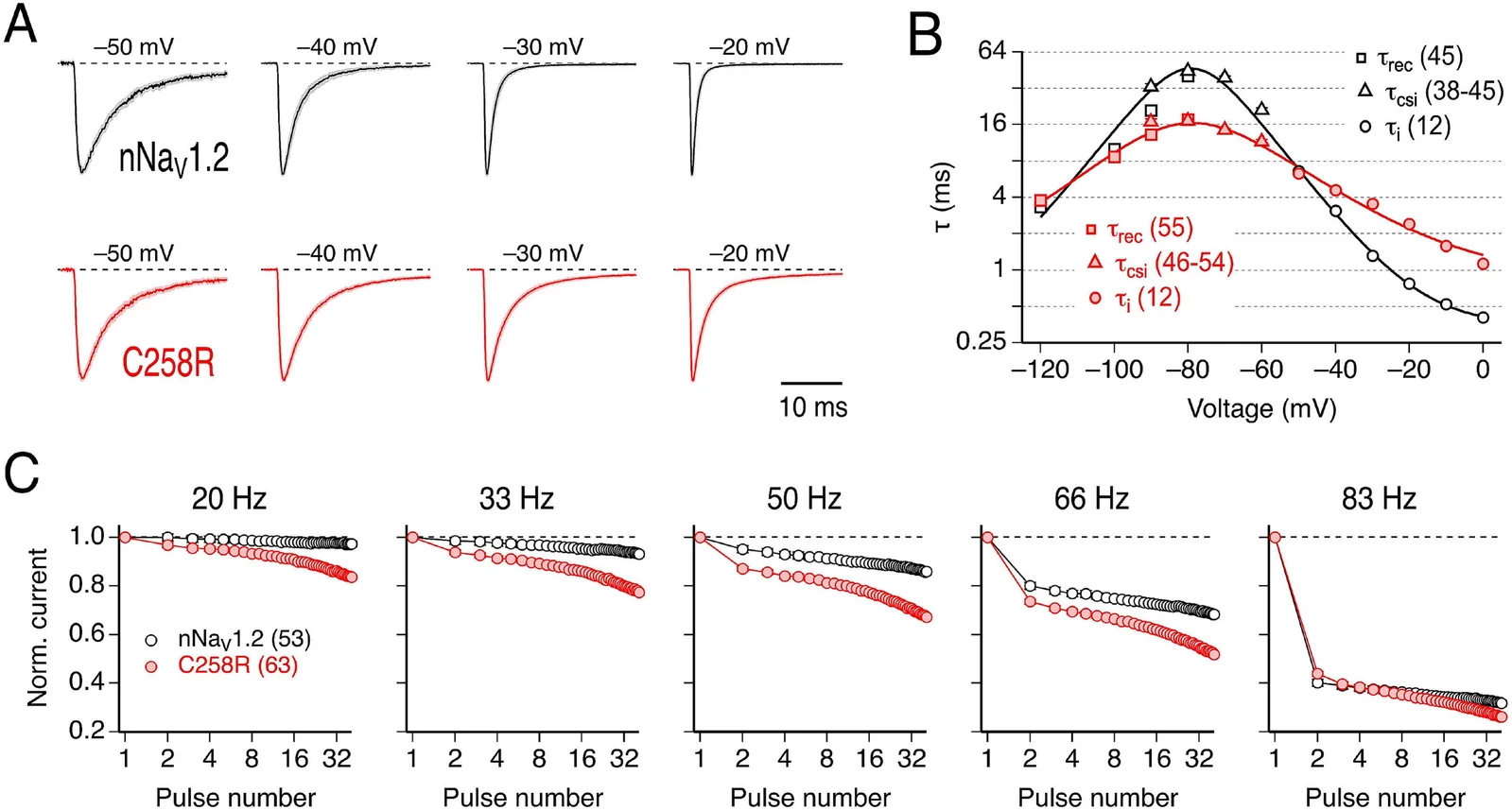
Kinetics of channel inactivation. **A**) Mean current traces (SEM indicated with shading), normalized to the peak current, for the indicated voltages and channel types (all *n* = 12). **B**) Inactivation time constant as a function of voltage. Depending on the voltage range, the inactivation time constant was measured with different protocols: squares denote time constants for the recovery from fast inactivation, triangles represent values obtained from closed-state inactivation, and circles are the inactivation time constants obtained from directly fitting Hodgkin & Huxley kinetics to the currents shown in A). The continuous curves are the results of fits (eq. [2]) assuming the inactivation occurs as a voltage-dependent single-step process yielding the values specified in section 3.2. **C**) Trains of peak inward currents measured during 10-ms depolarizations to −20 mV, normalized to the first pulse in a series and plotted against the pulse number. Recovery intervals at −120 mV of 40, 20, 10, 5, and 2 ms yielded frequencies of 20, 33, 50, 66, and 83 Hz, respectively. Straight lines connect data points for clarity. Data points in B) and C) are means ± SEM with *n* in parentheses.

Kinetics of inactivation were measured with three different protocols depending on the voltage range explored. The time courses of fast inactivation at voltages where channels are activated (−50 mV and higher) were analyzed by directly fitting a Hodgkin & Huxley gating function to the averaged and normalized current traces (Fig. 2A); the inactivation time constants are plotted in Fig. 2B as circles. At intermediate voltages, where closed-state inactivation occurs, two-pulse protocols were applied (Supplementary Fig. 1 A). The resulting time constants are shown in Fig. 2B as triangles. Finally, recovery from inactivation was measured with two-pulse protocols with increasing durations at the interpulse voltage between −120 and − 80 mV (Supplementary Fig. 1B): the resulting time constants are shown as squares in Fig. 2B.

The voltage dependences of all time constants characterizing the transitions between open (O) and inactivated (I) channel conformations were analyzed assuming a one-step reaction model of inactivation (O<–>I, eq. [2]) yielding *V*_h_ = −79.1 ± 0.3 mV and −80 ± 8 mV, τ_0_ = 0.34 ± 0.03 ms and 0.86 ± 0.09 ms, α_0_ = 10.8 ± 0.2 s^−1^ and 32 ± 4 s^−1^, *q* = 4.57 ± 0.01 e_0_ and 2.9 ± 0.5 e_0_, δ = 0.51 ± 0.01 and 0.54 ± 0.12 for wild-type and C258R channels, respectively.

Thus, mutation C258R has no major effect on the overall steady-state half-inactivation voltage (also see Figs. 1D) but mostly affects the gating kinetics (Fig. 2A, B). While at resting voltages the inactivation relaxation constant is faster than for the wild type, above a crossover at about −50 mV (where the channels start to activate), inactivation is slower. Consistent with the shallower voltage dependence of inactivation, it therefore appears as if the Na_V_1.2-C258R channels exhibit LoF properties at resting conditions and GoF properties on depolarization (Figs. 1D, 2A, B). This is also observed when channels are activated with voltage ramps. As shown in Supplementary Fig. 2, the waveform of such ramp-induced currents resulted in a relative current increase of mutant C258R over the wild type in the early (activation) and late (inactivation) phases of the ramp regardless of the speed of the voltage change.

After birth, the adult version of Na_V_1.2 is upregulated while nNa_V_1.2 is downregulated [37]. Thus, during early childhood, both variants are potentially relevant for the clinical outcome. We therefore also examined the voltage dependence and the basic kinetic parameters of Na_V_1.2 with and without the C258R mutation, and found similar functional shifts as for nNa_V_1.2 (Supplementary Fig. 3).

For rapid and repetitive channel activation, as occurs during high-frequency firing of neurons, trains of depolarizing pulses to −20 mV with varying interpulse episodes at −120 mV to yield pulse frequencies ranging between 20 and about 83 Hz were applied (Fig. 2C; for examples of current traces see Supplementary Fig. 4). At low to moderate pulse frequencies, the peak current of the mutant C258R consistently decreased more than that of the wild type. In particular after several depolarizing pulses, the stronger cumulative inactivation of the mutant becomes apparent. At very high frequencies, channels become significantly inactivated and the differences between wild type and mutant are only subtle.

**Fig. 3:**
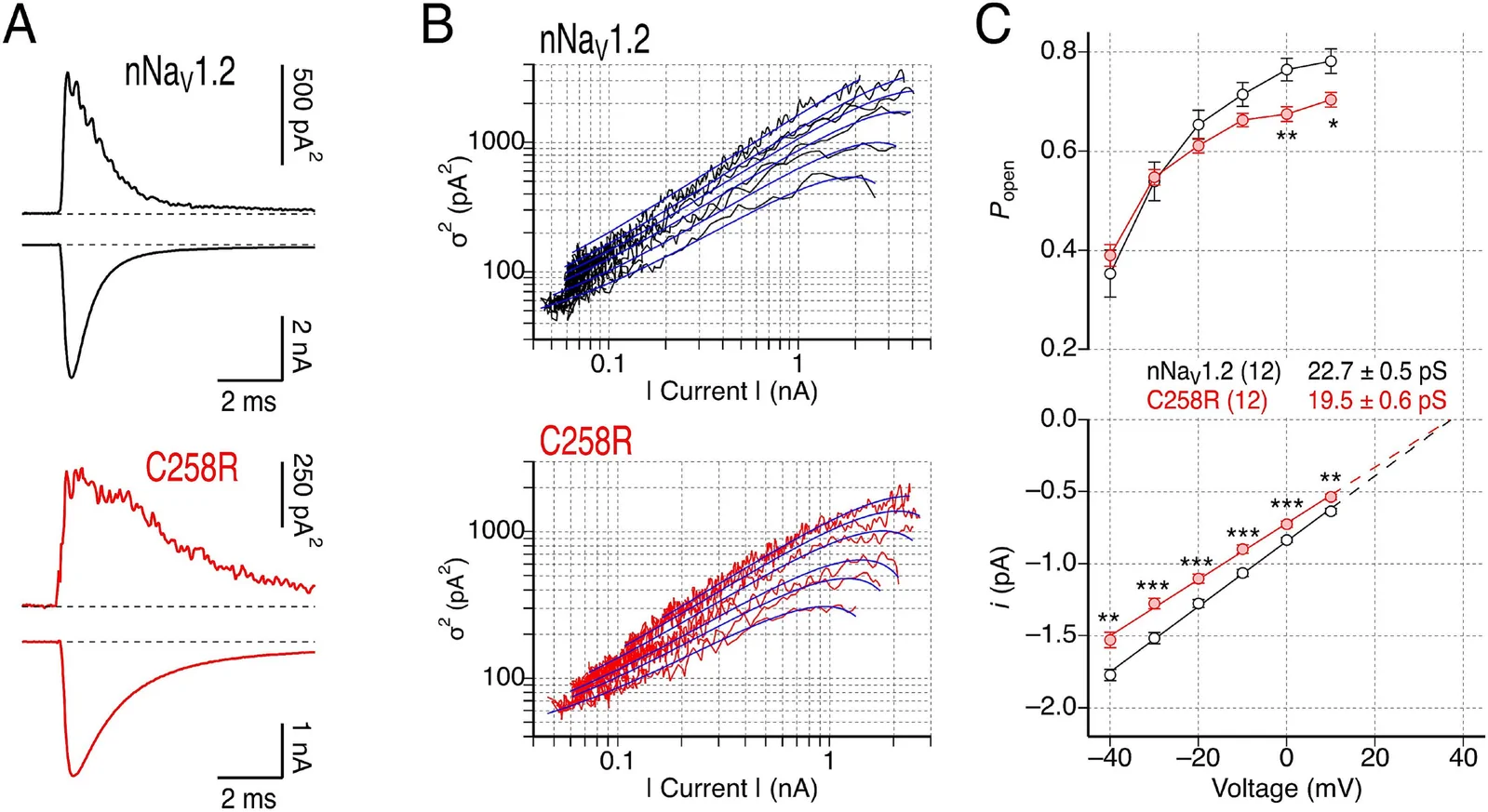
Estimation of single-channel parameters. **A**) Mean current traces (*bottom*) and mean ensemble variance (*top*) for 250 repetitive depolarizations to −20 mV for nNa_V_1.2 wild type (black) and the mutant C258R (red). **B**) Mean ensemble variance (σ^2^) as a function of leak-corrected mean current (|*I*|) for the falling phase of recordings as shown in A) for −40 mV through 10 mV in steps of 10 mV, from *top* to *bottom*. The superimposed blue traces are the results of global fits constraining the total number of channels, and considering the single-channel current (*i*) and the maximal open probability at the peak current (*P*_open_) as a function of voltage. **C**) Mean results of the global fits from 12 independent experiments each. Data points in the top panel (*P*_open_(*V*)) are connected by straight lines; the straight lines in the bottom panel (*i*(*V*)) are linear regressions yielding the single-channel conductance of 22.7 ± 0.5 pS for the wild type and 19.5 ± 0.6 pS for the mutant C258R, extrapolated to the reversal potential of 38 mV. Asterisks indicate statistically significant differences between pairs of data: *P < 0.05, **P < 0.01, ***P < 0.001. (For interpretation of the references to colour in this figure legend, the reader is referred to the web version of this article.)

The results shown in Fig. 1C indicate that one of the consequences of mutation C258R is a reduced current density in HEK293T cells. There can be numerous reasons for a smaller current density. For example, there may be a minor contribution due to slightly increased steady-state inactivation at −120 mV of the mutant compared to the wild type. Judging from the results shown in Fig. 1D, this may amount to at most 5% and would not fully explain a reduction in current density of nearly two-thirds. The mutation also may have consequences on translation, protein folding, or trafficking to the membrane – all factors that are not easily quantified. In addition, there may be an impact on the single-channel characteristics. Therefore, we performed non-stationary noise analysis based on the current fluctuations measured in many successive depolarizing steps to voltages ranging between −40 and 10 mV. Based on the resulting mean current and mean ensemble variances, the single-channel current and the maximally obtained open probability can be estimated [35]. Analysis of 24 independent cells, each with a total of 250 current traces per test voltage, yielded the results presented in Fig. 3: the single-channel conductance of the mutant was indeed smaller than that of the wild type. At −20 mV, *i.e.*, at the peak of the current-voltage relationship in Fig. 1C, this amounts to a peak current reduction of about 14%. At the same voltage, the maximal open probability was also slightly reduced by about 5%. These factors together sum up to about 25% current reduction, which is still less than observed for the current densities.

### 3.3 Action of antiseizure medications

**Fig. 4:**
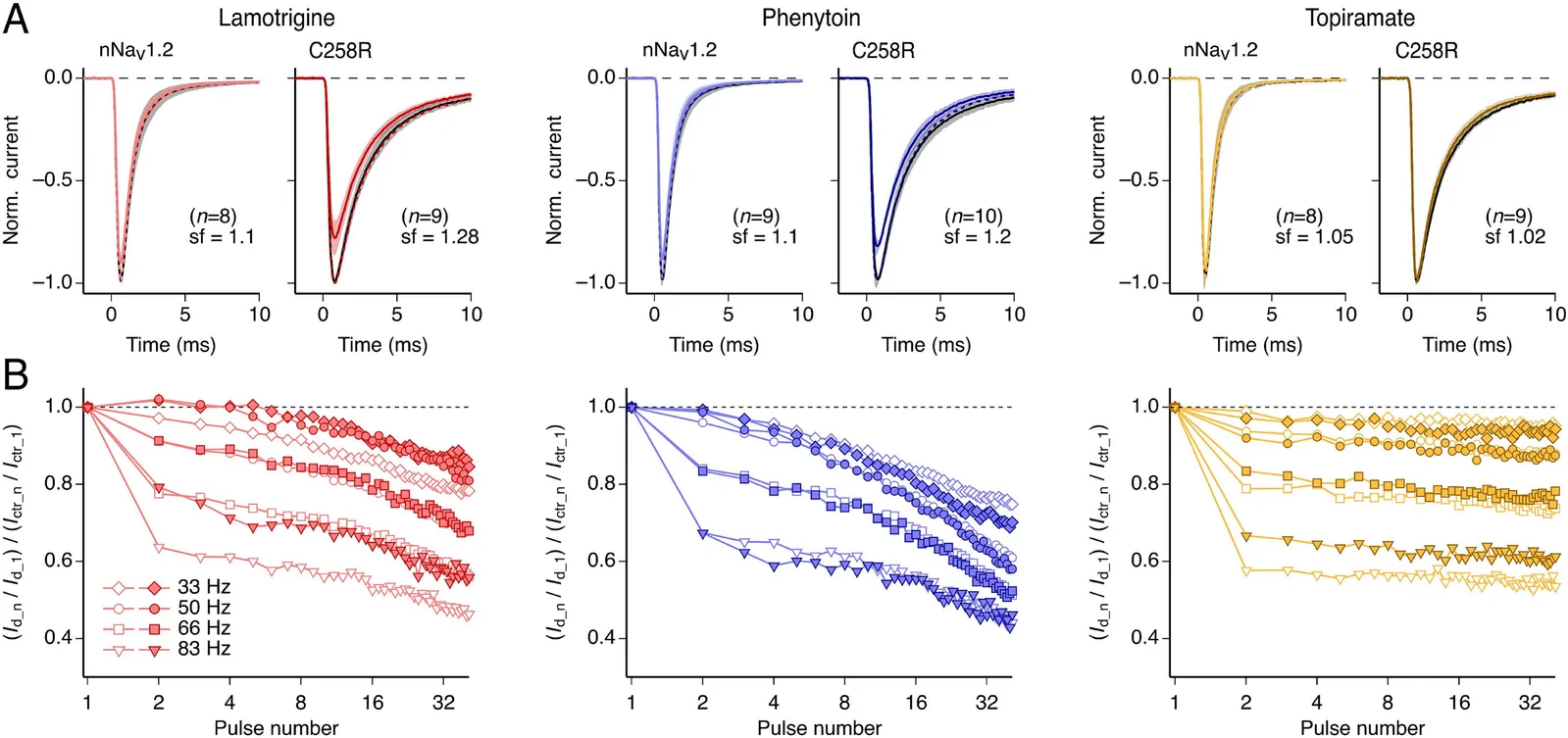
Use-dependent channel block by antiseizure medications that were part of the antiepileptic therapy. **A**) Superposition of mean current traces for nNa_V_1.2 wild type (*left*) and mutant C258R (*right*) at −20 mV before (black) and after application of 100 μM lamotrigine (*left*, red), phenytoin (*middle*, blue), or topiramate (*right*, orange). Error values (SEM) are indicated as shading, *n* is given in parentheses. Also superimposed are the mean traces after drug application, scaled to match the peak of the control (dashed lines); the corresponding scale factors (sf) are indicated. **B**) Average remaining peak current, relative to the values before drug application, for train protocols (as shown in Fig. 2C) for the wild type (open symbols) and mutant C258R (filled symbols) for the indicated pulse frequencies and drugs. Straight lines connect data points for clarity (*n* = 5–8). (For interpretation of the references to colour in this figure legend, the reader is referred to the web version of this article.)

The action of ASMs that were used for the treatment of the index patient (lamotrigine, phenytoin, topiramate) was assayed with respect to their blocking efficacy of nNa_V_1.2 wild-type and mutant channels. Drugs were focally applied to the cells while currents were recorded in the whole-cell recording mode. Extracellular and intracellular pH was 7.4. At a concentration of 100 μM, lamotrigine and phenytoin resulted in a steady-state current reduction of the wild type by about 10%, while the impact of topiramate was negligible; nNa_V_1.2-C258R was more strongly inhibited (28% for lamotrigine, 20% for phenytoin) (Fig. 4A). The time course of channel activation and inactivation was not affected. Since most Na_V_-active ASMs exhibit pronounced use-dependence, we also measured the progressive current decline during trains of depolarizations (as in Fig. 2C) with and without drugs. As shown in Fig. 4B, all three drugs block both the wild type and the mutant in a strongly use-dependent manner, to about 50% at the highest pulse frequency of 83 Hz. However, there were qualitative and quantitative differences among the drug actions. While for lamotrigine and phenytoin the fractional block increased over the full range of 41 pulses elicited, the use-dependent block by topiramate was almost saturated after the second pulse. Moreover, while the use-dependent block was about identical for the wild type and the mutant for phenytoin and topiramate, the fractional block of the mutant was consistently weaker than for the wild type when lamotrigine was applied.

Higher concentrations (300 μM) were not feasible for phenytoin due to the solubility limit. For topiramate the use-dependent block was not noticeably altered compared to 100 μM. For 300 μM lamotrigine, however, even low-frequency pulses (33 Hz) resulted in a cumulative channel inhibition of about 50%, while high-frequency pulses blocked the channels almost completely (Supplementary Fig. 5).

## 4 Discussion

The index patient presented with early-onset intractable seizures and severe developmental deficits. The epileptic part of the symptoms could be ameliorated with a combination of topiramate, phenytoin, and lamotrigine. Solely from the clinical picture no clear association with the molecular causes of the disease could be made. DNA sequencing disclosed a spontaneous mutation C258R in *SCN2A*, the gene encoding the α subunit of Na_V_1.2 channels.

When expressed in HEK293T cells and analyzed with electrophysiological methods, we found a wide spectrum of functional properties that differed between Na_V_1.2 and mutant C258R, not allowing a clear association with the major categories GoF or LoF. Based solely on the current density measured in HEK293T cells it appears as if the mutant produced fewer functional channels or channels with less open probability, totaling in an about 3-fold reduction of current density, while the single-channel conductance was only marginally reduced. However, current-density measurements strongly depend on the exact quantity and purity of the transfected DNA, protein production efficiency, and targeting – factors that not necessarily replicate the situation in a patient. Based on current kinetics at depolarization exceeding −40 mV, the slower inactivation kinetics of C258R suggest a GoF phenotype. This excess current may be more than overcompensated by faster closed-state inactivation below −50 mV (Fig. 2B), a more pronounced steady-state inactivation around resting membrane potential (Fig. 1D), and a slightly reduced single-channel conductance and maximum open probability (Fig. 3). Moreover, repetitive stimulation appears to gradually shift C258R channels to an inactivated state (Fig. 2C). Collectively, these individual channel properties suggest a mixed GoF/LoF phenotype, mainly by alteration of channel inactivation.

Our results are compatible with recent studies reporting three epilepsy-associated mutations in Na_V_1.2 that also affect amino acids located in S5 of domain I: M252V, V261M, A263V [19], [38]. The mutations selectively affected the inactivation properties of Na_V_1.2, compatible with an overall GoF phenotype. While all three alterations increased the fraction of persistent, non-inactivating current components and accelerated the channel's recovery from fast inactivation, V261M and A263V additionally caused a slowdown of fast inactivation kinetics. In this context, mutation Q270K in DI/S5 of Na_V_1.4 channels also seems worth mentioning [39] because it causes *Paramyotonia congenita* and disturbs channel inactivation, mainly characterized by a faster recovery from inactivated states, substantially slowed inactivation kinetics, and a depolarized voltage dependence of steady-state inactivation. Rapid inactivation of Na_V_ channels is mediated by an interaction of the intracellular DIII/IV linker (Fig. 1A) with the channel pore, a process that is coupled to the movement of the voltage sensor in DIV [40]. The voltage sensor itself is composed of transmembrane segments S1-S4 in DIV, with several positively charged side chains in S4 serving as gating charges. The three-dimensional structure of Na_V_1.4 [41] predicts that amino acid position 258 in DI/S5 of Na_V_1.2 (Fig. 1A) is located less than 10 Å away from S4 in DIV, allowing for both steric and electrostatic interactions between both epitopes, potentially affecting voltage-sensor movements and channel inactivation.

Unlike Na_V_1.1, which is highly expressed in inhibitory interneurons [42], Na_V_1.2 is found predominantly in excitatory [43] and glutamatergic neurons [10] of the CNS. Patients with Na_V_1.2 GoF variants typically present with early-onset epilepsies and often respond well to sodium-channel blockers, particularly phenytoin and carbamazepine. In contrast, LoF variants are associated with late-onset seizures that respond poorly to sodium-channel block or may even be exacerbated by this medication [11]. Thus, the early onset of seizures in our patient and his favorable response to phenytoin suggest that his seizures are largely driven by GoF features of C285R channels, mediated by impaired channel inactivation. The therapeutic advantage of phenytoin was also reflected in our *in vitro* electrophysiological recordings: of the three ASMs studied, phenytoin was the most effective at suppressing repeated activation of C258R mutant channels.

The classical ASMs phenytoin and lamotrigine are tricyclic compounds that appear to interact with residues located in the S6 helix in DIV, largely overlapping with the channel's local anesthetics binding site [44], [45]. However, different forms of Na_V_-dependent epilepsies exhibit different responses to phenytoin and lamotrigine, even though both drugs interfere primarily with fast Na_V_ channel inactivation [46], [47]. The reasons for their different efficacy profiles may be attributed to molecular and physiological mechanisms underlying Na_V_-related epileptic syndromes, as well as to the distinct chemical and pharmacokinetic properties of each compound. In line with the observed diversity of functions, a recent molecular simulation study revealed that for lamotrigine a clear binding site cannot be defined, as it mainly uses the fenestrations, hydrophobic cavities between individual channel domains, to interfere with the channel [48]. In addition, neither phenytoin nor lamotrigine are specific Na_V_-channel blockers. For example, although classified as typical state-dependent inhibitor of Na_V_ channels, phenytoin has also weak secondary effects on L- and T-type voltage-gated calcium (Ca_V_) channels and NMDA receptors [49], [50]. By contrast, lamotrigine combines Na_V_ channel block with the inhibition of presynaptic N- and P/Q-type Ca_V_ channels and a strong suppression of glutamate release [51], [52], explaining its widespread use as ASM and mood stabilizer.

Topiramate is a broad-spectrum multi-target ASM with a unique polypharmacological profile. In addition to stabilizing inactivated states of Na_V_ channels [53], [54], it affects also GABAergic and glutamatergic pathways: it enhances GABAergic neurotransmission by acting as positive allosteric modulator of GABA-A receptors, inhibits AMPA and kainite receptors, and suppresses the activity of L- and N-type Ca_V_ channels, all of which reduce hyperexcitable states of the CNS [55], [56], [57]. Furthermore, topiramate is a weak inhibitor of carbonic anhydrase isoforms II and IV, potentially contributing to its anticonvulsant activity [58]. A recently reported cryo-EM structure of topiramate-bound Ca_V_3.2 channels revealed that the drug occupies an intracellular hydrophobic pocket located at the interface between channel domains I and II, where it likely acts as an allosteric modulator of channel gating [59]. Na_V_ channels harbor a similar hydrophobic cavity, which is located at the interface between DII and DIII, positioning it in close proximity to the channels IFM inactivation motif. Nevertheless, systematic electrophysiological recordings of cultured mouse spinal cord neurons demonstrated that topiramate is markedly less effective than phenytoin or lamotrigine in suppressing recurrent Na_V_-mediated action potentials. The data support the view that inhibition of Na_V_ channel activity contributes only modestly to the overall pharmacological profile of topiramat and that Na_V_-independent mechanisms are the predominant mediators of its anticonvulsant activity [60].

Besides displaying typical epilepsy symptoms, the patient also suffered from developmental retardation and ASD-like symptoms. The latter might be associated with LoF of Na_V_1.2 as one of the leading congenital indicators for ASD (*e.g.*, [26]). Since such symptoms were unaltered with the administration of the above-mentioned medication, it is conceivable that in early developmental steps the Na_V_1.2 LoF aspects dominate, while at later stages GoF of the same channel may express a potentially druggable epileptogenic potential. Distinct expression patterns of Na_V_1.2 in different brain regions may add to the complex clinical phenotype and its limited response to pharmacological interventions.

## 5 Conclusion

We demonstrated that the Na_V_1.2-C258R variant results in a mixed LoF/GoF phenotype, with the GoF component most likely accounting for the epilepsy symptoms that were difficult to treat. For *SCN2A*-related developmental and epileptic encephalopathy type 11 caused by the C258R variant, both the clinical presentation and the experimental data support the use of phenytoin and topiramate — antiseizure medications of the extended spectrum — as first-line therapy within the framework of personalized medicine.

## CRediT authorship contribution statement

**Alina Köppel:** Writing – review & editing, Investigation. **Heike de Vries:** Writing – review & editing, Supervision, Investigation. **Mahdi Jamili:** Writing – review & editing, Methodology, Investigation, Formal analysis. **Christina Stanke:** Investigation. **Ulrich Brandl:** Supervision, Conceptualization. **Enrico Leipold:** Writing – review & editing, Supervision, Investigation, Formal analysis, Conceptualization. **Stefan H. Heinemann:** Writing – original draft, Visualization, Supervision, Project administration, Funding acquisition, Formal analysis, Data curation, Conceptualization.

## Ethics Approval statement

The study was approved by the Ethics Committee of the University Hospital Jena (File number: 2025–4055-BO-D).

## Funding

This work was supported by the Interdisciplinary Center for Clinical Research Jena (AK) Jena and the 10.13039/100014370Simons Foundation Autism Research Initiative (SHH, 705944SH).

## Declaration of competing interest

The authors declare that they have no known competing financial interests or personal relationships that could have appeared to influence the work reported in this paper.
