# Supplementary material for: Functional and pharmacological characterization of the SCN2A variant p.C258R with mixed gain and loss of function causing developmental and epileptic encephalopathy type 11
Source: Epilepsy Behav Rep. 2026 Sep 11;36:100897. doi: 10.1016/j.ebr.2026.100897 (PMC13595215; doi:10.1016/j.ebr.2026.100897)
Supplement: Supplementary file 1 [file mmc1.pdf]

Functional and pharmacological characterization of the *SCN2A* variant p.C258R with mixed gain- and loss-of-function causing developmental and epileptic encephalopathy type 11

Supplementary Information

Alina Köppel[a,b#], Heike de Vries[b#], Mahdi Jamili[a], Christina Stanke[c], Ulrich Brandl[b], Enrico Leipold[a,c], and Stefan H. Heinemann[a*]

[a] Center for Molecular Biomedicine, Department of Biophysics, Friedrich Schiller University Jena and Jena University Hospital, Jena, Germany.

[b] Department of Neuropediatrics, Jena University Hospital, Jena, Germany.

[c] Department of Anesthesiology and Intensive Care, Lübeck University Hospital, Center for Brain, Behavior and Metabolism (CBBM), University of Lübeck, Germany.

# Shared first authors

*Corresponding author:

Center for Molecular Biomedicine, Department of Biophysics, Friedrich Schiller University Jena & Jena University Hospital, Hans-Knöll-Str. 2, 07745 Jena, Germany.

stefan.h.heinemann@uni-jena.de ORCID: 0000-0002-4144-0251

Supplementary Tables

Supplementary Table 1. Parameters characterizing the voltage dependence and the kinetics of $nNa_V1.2$ and $Na_V1.2$ along with the C258R mutants. Voltage dependence of activation: V_m, k_m (Eq. 1); voltage dependence of inactivation with 0.5 s and 10 s conditioning: $V_{h0.5s}$, $k_{h0.5s}$, V_{h10s}, k_{h10s}; time constant for the recovery from inactivation (τ_{rec}) and closed-state inactivation (τ_{CSI}) and the indicated voltages. Data are means ± SEM.

	$nNa_V1.2$	*n*	C258R	*n*
V_m (mV)	-46.8 ± 0.5	81	-50.2 ± 0.5	89
k_m (mV)	11.43 ± 0.16		11.41 ± 0.13	
$V_{h0.5s}$ (mV)	-77.8 ± 0.5	53	-81.3 ± 0.6	62
$k_{h0.5s}$ (mV)	5.77 ± 0.12		10.3 ± 0.17	
V_{h10s} (mV)	-79.8 ± 0.4	53	-84.7 ± 0.6	62
k_{h10s} (mV)	5.32 ± 0.10		8.95 ± 0.11	
τ_{rec} (ms), -120 mV	3.33 ± 0.12	45	3.78 ± 0.12	53
-100 mV	10.0 ± 0.4	45	8.67 ± 0.28	53
-90 mV	21.0 ± 1.1	45	13.2 ± 0.4	53
-80 mV	40.5 ± 1.7	45	17.6 ± 0.5	53
τ_{CSI} (ms), -60 mV	21.1 ± 1.0	45	11.5 ± 0.6	54
-70 mV	39.2 ± 1.2	45	14.4 ± 0.4	54
-80 mV	44.3 ± 1.5	45	17.1 ± 0.7	53

	$Na_V1.2$	*n*	C258R	*n*
V_m (mV)	-34.7 ± 0.5	24	-41.1 ± 0.8	21
k_m (mV)	11.8 ± 0.4		12.1 ± 0.2	
$V_{h0.5s}$ (mV)	-72.6 ± 0.5	24	-74.3 ± 1.2	20
$k_{h0.5s}$ (mV)	5.76 ± 0.18		10.4 ± 0.3	
V_{h10s} (mV)	-75.3 ± 0.6	24	-78.6 ± 1.1	21
k_{h10s} (mV)	5.39 ± 0.20		8.91 ± 0.25	
τ_{rec} (ms), -120 mV	2.21 ± 0.11	24	2.44 ± 0.17	21
-100 mV	6.2 ± 0.3	24	5.4 ± 0.4	21
-90 mV	12.0 ± 0.6	24	8.4 ± 0.6	21
-80 mV	25.8 ± 1.2	24	12.6 ± 0.7	21

τ_{CSI} (ms), -60 mV	27.6 ± 1.6	24	12.0 ± 0.8	18
-70 mV	41.3 ± 1.9	24	14.8 ± 0.8	18
-80 mV	35.4 ± 1.6	24	16.1 ± 1.1	18

Supplementary Figures

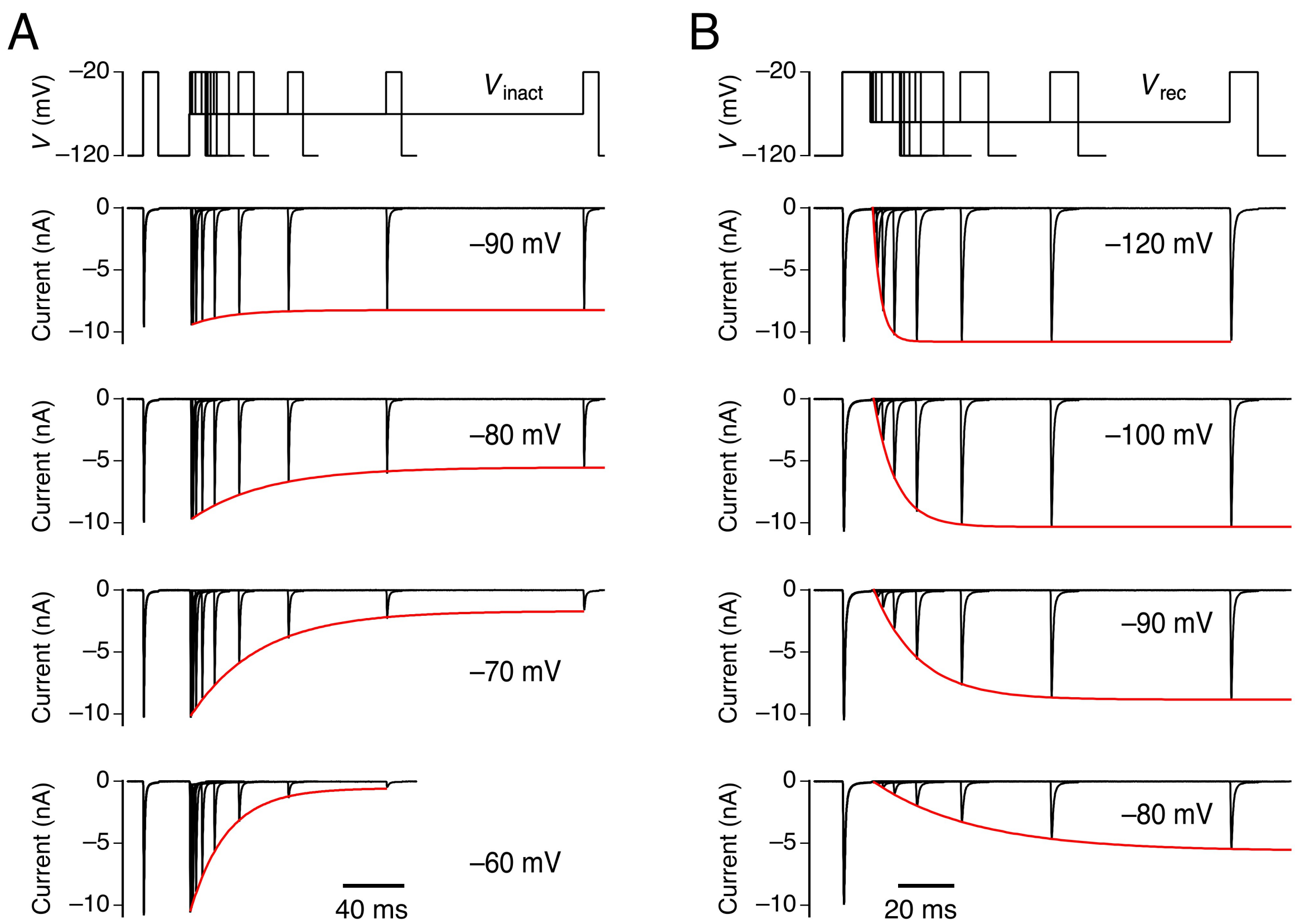

Supplementary Figure 1. Measurement of closed-state inactivation and recovery from inactivation. **A**) Superimposed whole-cell current traces of $nNa_V1.2$ channels to assess the kinetics of closed-state inactivation at the indicated voltages. The pulse protocol, exemplified here for the inactivation voltage of –70 mV, is indicated in the top panel. Single-exponential fits (red) are superimposed to match the kinetics of peak current decrease.
B) Current data from the same cell as in A) for the determination of the recovery from inactivation at the indicated voltages with superimposed single-exponential fits. The pulse protocol exemplifies the recovery voltage of –80 mV.

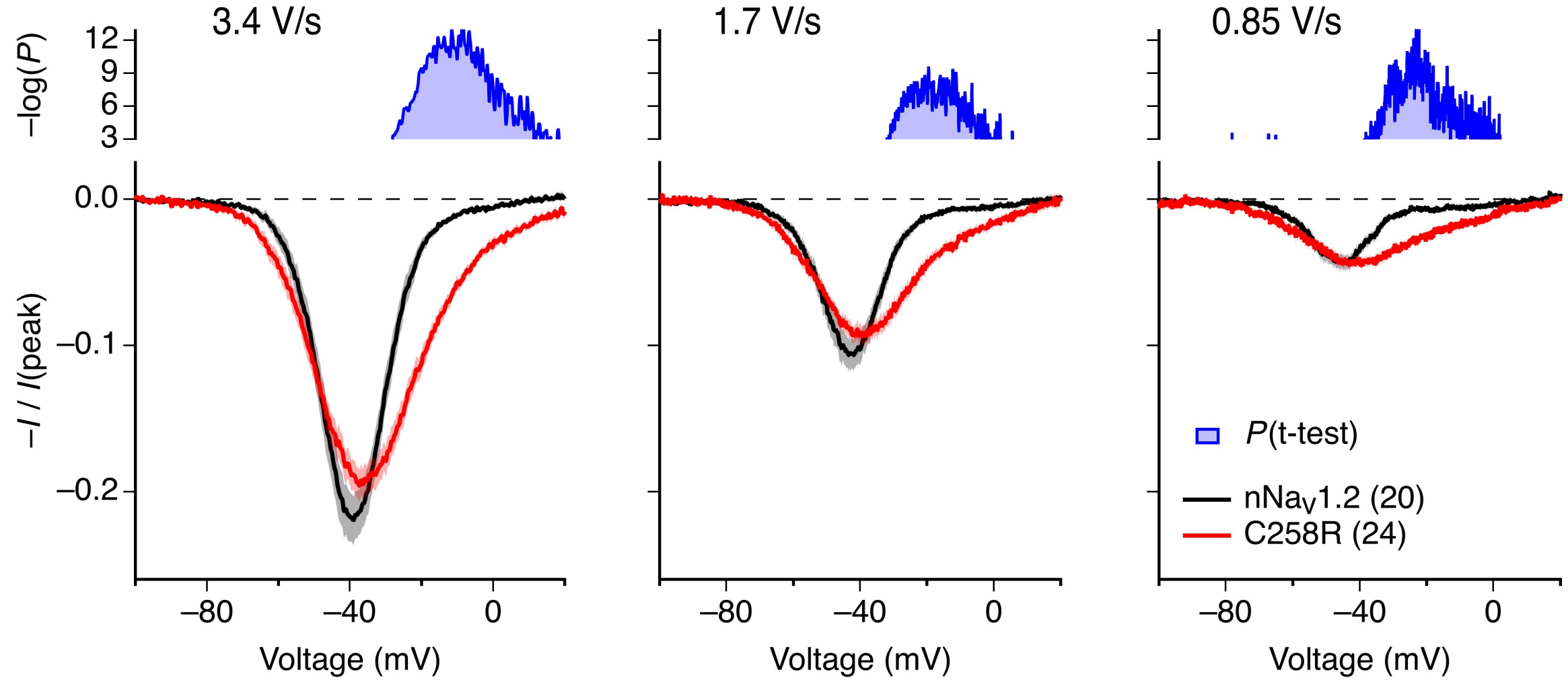

Supplementary Figure 2. Current responses to voltage ramps. Mean whole-cell current traces (SEM in shading, *n* in parentheses) of wild-type and mutant C258R nNa$_V$1.2 channels in response to stimulation with voltage ramps (–120 to 50 mV in 50, 100, and 200 ms, corresponding to slopes of 3.4, 1.7, and 0.85 V/s). The data are normalized to the mean inward current obtained during current-voltage recordings, as shown in Fig. 1B. The results of non-paired t-tests for individual sample points are indicated as –log(*P*), thus marking voltages within the ramps where the significance is greater than $P = 0.001$. The integrals between –100 and 20 mV were consistently larger for the mutant by factors of 1.41 (3.4 V/s), 1.39 (1.7 V/s), and 1.72 (0.85 V/s).

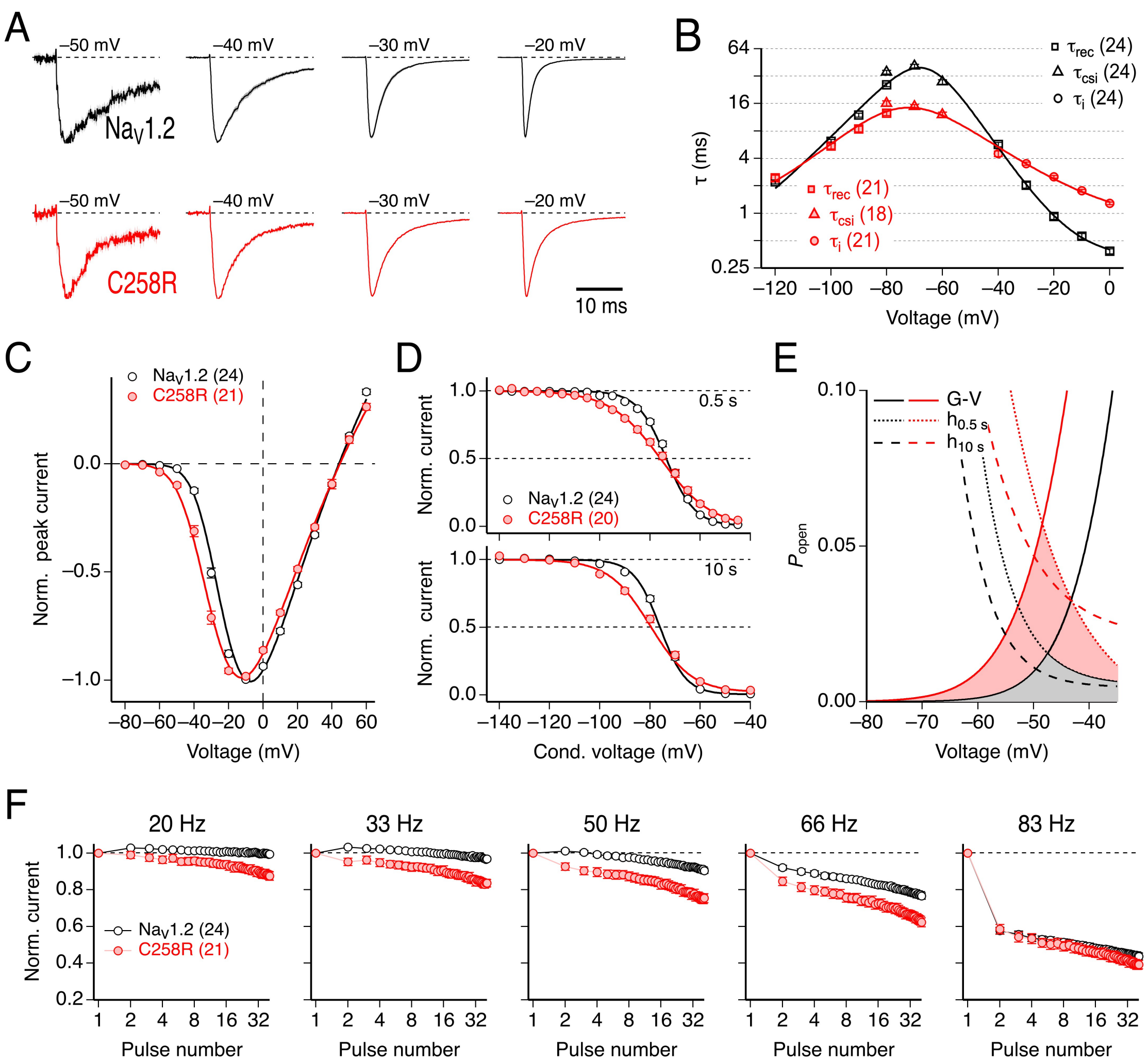

Supplementary Figure 3. Functional properties of adult Na$_V$1.2 and mutation C258R. **A**) Mean current traces (SEM indicated with shading), normalized to the peak current, for the indicated voltages and channel types (all *n* = 20). **B**) Inactivation time constant as a function of voltage. Depending on the voltage range, the inactivation time constant was measured with different protocols: squares denote time constants for the recovery from fast inactivation, triangles represent values obtained from closed-state inactivation, and circles are the inactivation time constants obtained from directly fitting Hodgkin & Huxley kinetics to the currents shown in A). The continuous curves are the results of fits (eq. [2]) assuming the inactivation occurs as a voltage-dependent single-step process yielding the following values: V_h = –66.2 ± 0.7 mV and –75.5 ± 1.8 mV, τ_0 = 0.32 ± 0.03 ms and 0.76 ± 0.10 ms, α_0 = 12.9 ± 0.4 s^{-1} and 36.6 ± 1.2 s^{-1}, *q* = 4.5 ± 0.1 e_0 and 2.9 ± 0.1 e_0, δ = 0.59 ± 0.01 and 0.54 ± 0.02 for wild-type Na$_V$1.2 and C258R channels, respectively. **C**) Peak currents normalized to the maximal inward current as a function of voltage. The continuous curves are data fits according to eq. [1]. **D**) Mean maximal currents at –20 mV as a function of voltage of a 500-ms (*top*) or 10-s (*bottom*) prepulse, normalized to the values obtained at –120 mV. Curves are the results of Boltzmann-type fits (eq. [3]). **E**) Window current for Na$_V$1.2 and mutant C258R, estimated from the voltage-dependent activation (solid line) and steady-state inactivation with 0.5 s or 10 s conditioning (dotted and dashed lines, respectively). **F**) Trains of peak inward currents measured during 10-ms depolarizations to –20 mV, normalized to the first pulse in a series and plotted against the pulse number. Recovery intervals at –120 mV of 40, 20, 10, 5, and 2 ms yielded frequencies of 20, 33, 50, 66, and 83 Hz,

respectively. Straight lines connect data points for clarity. Data points in B), C), D), and F) are means ± SEM with *n* in parentheses.

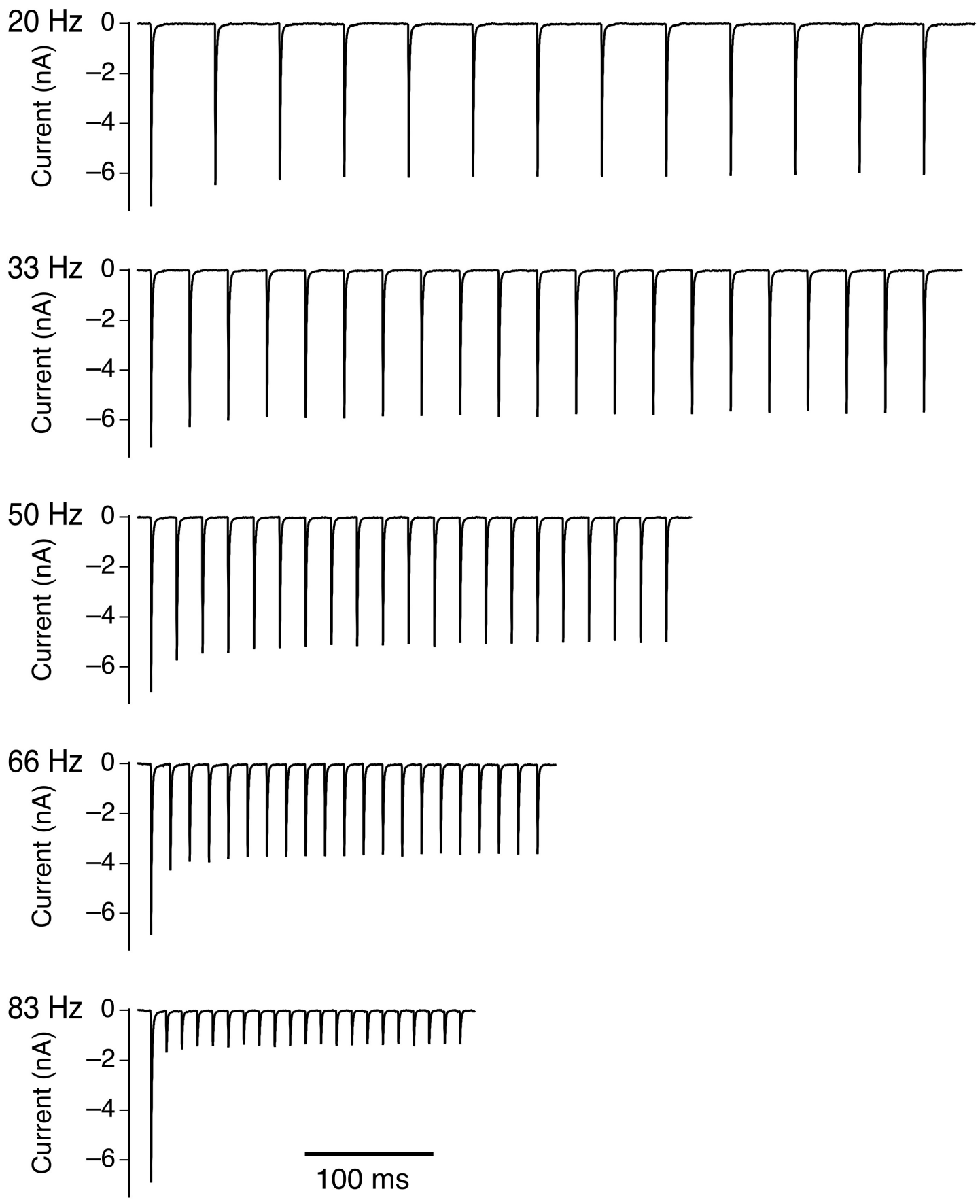

Supplementary Figure 4. Pulse frequency-dependent channel inactivation. Whole-cell current traces of $nNa_V1.2$ channels subjected to repetitive 10-ms depolarizations to –20 mV from –120 mV. The intervals at –120 mV were 40, 20, 10, 5, and 2 ms to yield pulse frequencies of 20, 33, 50, 66, and 83 Hz, respectively.

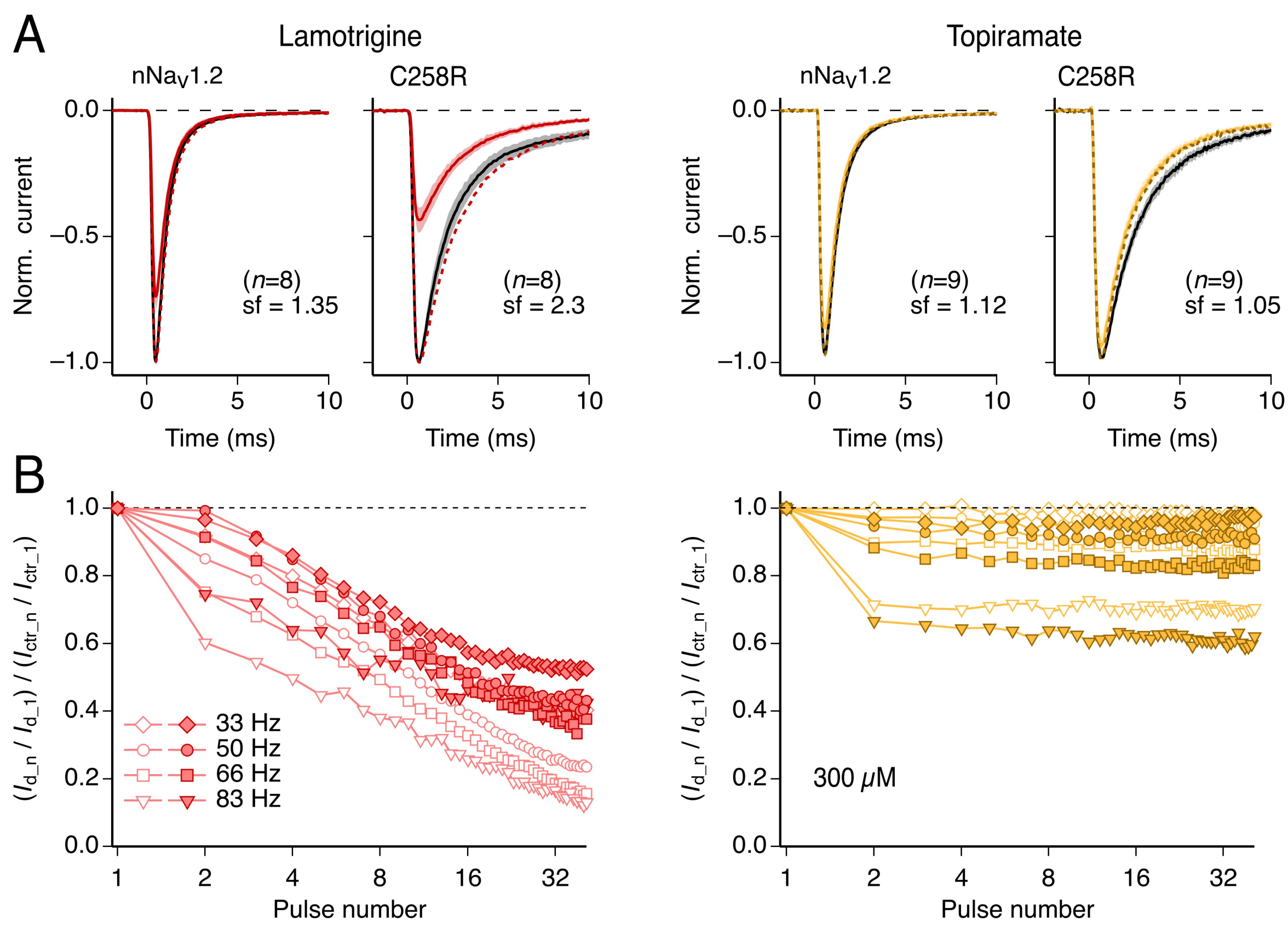

Supplementary Figure 5. Use-dependent channel block by lamotrigine and topiramate. **A**) Superposition of mean current traces for $nNa_V1.2$ wild type (*left*) and mutant C258R (*right*) at –20 mV before (black) and after application of 300 µM lamotrigine (*left*, red) or topiramate (*right*, orange). Error values (SEM) are indicated as shading. Also superimposed are the scaled mean traces after drug application to match the peak of the control (dashed lines); the corresponding scale factors (sf) are indicated. **B**) Average remaining current, relative to the values before drug application, for train protocols (as shown in Fig. 2C) for the wild type (open symbols) and mutant C258R (filled symbols) for the indicated pulse frequencies. Straight lines connect data points for clarity (*n* = 8-9).
